# Unsupervised machine learning for identifying important visual features through bag-of-words using histopathology data from chronic kidney disease

**DOI:** 10.1038/s41598-022-08974-8

**Published:** 2022-03-22

**Authors:** Joonsang Lee, Elisa Warner, Salma Shaikhouni, Markus Bitzer, Matthias Kretzler, Debbie Gipson, Subramaniam Pennathur, Keith Bellovich, Zeenat Bhat, Crystal Gadegbeku, Susan Massengill, Kalyani Perumal, Jharna Saha, Yingbao Yang, Jinghui Luo, Xin Zhang, Laura Mariani, Jeffrey B. Hodgin, Arvind Rao, the C-PROBE Study

**Affiliations:** 1 Department of Computational Medicine and Bioinformatics, University of Michigan, Ann Arbor, MI USA; 2 Department of Pathology, University of Michigan, Ann Arbor, MI USA; 3 Department of Internal Medicine, Nephrology, University of Michigan, Ann Arbor, MI USA; 4 Department of Pediatrics, Pediatric Nephrology, University of Michigan, Ann Arbor, MI USA; 5 Department of Internal Medicine, Nephrology, St. Clair Nephrology Research, Detroit, MI USA; 6 Department of Internal Medicine, Nephrology, Wayne State University, Detroit, MI USA; 7 Department of Internal Medicine, Nephrology, Cleveland Clinic, Cleveland, OH USA; 8 Department of Pediatrics, Pediatric Nephrology, Levine Children’s Hospital, Charlotte, NC USA; 9 Department of Internal Medicine, Nephrology, Department of JH Stroger Hospital, Chicago, IL USA; 10 Department of Biostatistics, University of Michigan, Ann Arbor, MI USA; 11 Department of Radiation Oncology, University of Michigan, Ann Arbor, MI USA; 12 Department of Biomedical Engineering, University of Michigan, Ann Arbor, MI USA

**Keywords:** Computational biology and bioinformatics, Computational models, Chronic kidney disease

## Abstract

Pathologists use visual classification to assess patient kidney biopsy samples when diagnosing the underlying cause of kidney disease. However, the assessment is qualitative, or semi-quantitative at best, and reproducibility is challenging. To discover previously unknown features which predict patient outcomes and overcome substantial interobserver variability, we developed an unsupervised bag-of-words model. Our study applied to the C-PROBE cohort of patients with chronic kidney disease (CKD). 107,471 histopathology images were obtained from 161 biopsy cores and identified important morphological features in biopsy tissue that are highly predictive of the presence of CKD both at the time of biopsy and in one year. To evaluate the performance of our model, we estimated the AUC and its 95% confidence interval. We show that this method is reliable and reproducible and can achieve 0.93 AUC at predicting glomerular filtration rate at the time of biopsy as well as predicting a loss of function at one year. Additionally, with this method, we ranked the identified morphological features according to their importance as diagnostic markers for chronic kidney disease. In this study, we have demonstrated the feasibility of using an unsupervised machine learning method without human input in order to predict the level of kidney function in CKD. The results from our study indicate that the visual dictionary, or visual image pattern, obtained from unsupervised machine learning can predict outcomes using machine-derived values that correspond to both known and unknown clinically relevant features.

## Introduction

Chronic Kidney Disease (CKD) is the 9th leading cause of death in the U.S. and results in more deaths than either breast cancer or prostate cancer^1^. CKD entails the gradual loss of kidney function and progressive loss of normal structure identified on kidney biopsy. It is defined by glomerular filtration rate (GFR) less than 60 ml/min/1.73 m^2^ for 3 months or more, and/or loss of protein in the urine and is associated with certain structural abnormalities in the kidney. The degree of kidney dysfunction is associated with increased mortality and risk of heart disease^2,3^. Thus, early detection with accurate diagnosis is critical to slow the risk of progression to kidney failure^4^. Currently, creatinine level in the blood, which can be used to estimate GFR, and protein in the urine are the best noninvasive measures of kidney function and risk of progression^5,6^. However, creatinine and other noninvasive surrogates for GFR have several limitations and are not accurate at higher levels of kidney function^7^. Kidney biopsy samples may provide further prognostic information such as degree of glomerular sclerosis and interstitial fibrosis^8^. These are often visually estimated, and interpretation may vary among pathologists. To overcome substantial inter-observer variability, computer-aided algorithms can help to provide an objective manner of kidney assessment.

Recently, with the growing availability of whole-slide digital scanners, digital pathology has become increasingly common in clinical research^9^. Digitizing pathology allows researchers and clinicians to leverage computer-aided algorithms to capture and quantify biologically meaningful information from complex whole slide images (WSI). These algorithms facilitate more standardized quantification and greater reproducibility of descriptive findings on biopsy slides^10–14^. The advent of artificial intelligence including deep learning and machine learning algorithms has optimized our ability to process and analyze the large amounts of data provided by whole-slide digital scanners. These methods are commonly used in computer vision, radiology, and oncology^15–22^. Several deep learning and machine learning approaches have been used in renal pathology. Convolutional neural networks (CNN) have been applied to WSI for distinguishing sclerosed from non-sclerosed glomeruli^23,24^. Hermsen et al. used deep learning to segment several histologic structures on PAS stained tissue, including glomeruli, proximal and distal tubules, atrophic tubules and blood vessels^25^. The ability to segment these structures provides opportunities for standardized approaches to histologic quantification and diagnosis^26^. Kolachalama et al. demonstrated that CNN models can outperform the pathologist-estimated fibrosis score across the classification tasks, including CKD stage and renal survival^27^.

To date, deep learning algorithms applied to renal histology have focused on supervised approaches. A supervised algorithm requires the use of a labeled training set, which may be a cumbersome task. The present study utilizes an unsupervised machine learning method called bag-of-words to identify important patterns or features that are associated with the level of kidney function and risk of progression. An unsupervised machine learning algorithm learns from an unlabeled dataset and automatically finds structure or pattern in the data by extracting useful features^28^. The bag-of-words model is a known computer vision classifier which was originally used in natural language processing and information retrieval. It is commonly used in document classification tasks where the frequency or occurrence of each word is used as a feature for training a classifier^29^. Each document is represented as a “bag” and can be identified by a pattern or frequency of “words”/features extracted from the document. Its utility has been demonstrated in predicting survival in gliomas^19^. In that study, the bag-of-words model identified key “words” or “phenotypes” (i.e., structurally similar image segments) in glioma biopsy slides for predicting survival. In addition to obviating the need for a labeled training set, the bag-of-words model identified clinically relevant histologic features which were correlated with survival. This was regardless of the tumor type, which is the typically used to classify survival. Some of the key image phenotypes also correlated with disease-associated molecular signaling activity. The bag-of-words model in this setting has the potential to identify determinants of survival that can be accessories to traditional clinical models.

In this study, we developed a bag-of-words (BoW) model to extract key features or visual words from WSI of renal biopsies, and then spatially encoded the original histopathology images using these words^30,31^. The BoW algorithm identifies important image segments that correlate with kidney function at the time of biopsy as well as predicting loss of function at one year. The BoW algorithm allows pathologists to inspect these key image segments for clinically significant data. As opposed to traditional deep learning approaches, where an algorithm learns from data labeled by pathologist who already have a structured framework for classifying disease, this unsupervised approach can hypothetically allow for novel ways to classify diseases and potentially identify more useful frameworks to understand and prognosticate disease. The *hypothesis* of this study is that unsupervised machine learning algorithms, without human input, can identify novel predictors of kidney function and renal prognosis.

## Methods

### Data collection

The study population included patients enrolled in the C-PROBE cohort, a multicenter cohort of patients with CKD established under auspices of the George O’Brien Kidney Center at the University of Michigan (https://kidneycenter.med.umich.edu/clinical-phenotyping-resource-biobank-core), aimed at collecting high-quality data and biosamples for translational research approved by the Institutional Review Boards of the University of Michigan Medical School (IRBMED) with approval number HUM00020938. C-PROBE enrolls patients at the time of clinically indicated biopsy and follows them with phenotypic data prospectively. The cohort includes an ethnically diverse population with a wide range of CKD stage and diverse etiologies of kidney disease. The informed consent was obtained from all subjects and/or their legal guardians.

A total of 107,471 histopathology images (256 × 256 pixels) were obtained from 161 biopsy cores from 57 patients in the form of trichrome-stained slides. This study was conducted and carried out in accordance with relevant guidelines and regulations. The Chronic Kidney Disease Epidemiology Collaboration (CKD-EPI) formula was used to calculate estimated glomerular filtration rate (eGFR)^7,32^.

**Figure 1:**
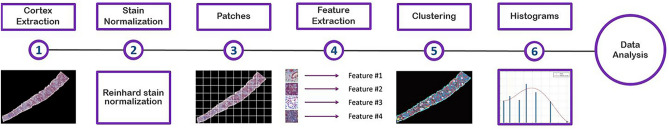
Workflow for the unsupervised learning using a bag-of-words paradigm. In step (1) the cortex part of the biopsy sample was used; (2) the Reinhard stain color normalization method applied; (3) each biopsy sample image was tiled into 256 × 256 pixel patches; (4) we extracted features from each patch using the transfer learning method in deep learning; (5) unsupervised machine learning algorithms called K-means clustering was applied; and finally (6) a histogram representation for each biopsy sample was created to describe the distribution of each type of cluster at the patient level.

The overall workflow for the unsupervised machine learning using a bag-of-words paradigm is shown in Fig. 1. First, we used the cortex part of the biopsy sample on a whole-slide-image and then we removed the background by making it black. Then, all tissue samples in trichrome-stained images were normalized using the Reinhard stain color normalization method^33^, which matches the color distribution of an image to that of a target image and therefore is an important step in clustering and classification tasks, so that clustering was not driven by differences in staining variations. And then, each normalized biopsy sample image was tiled into 256 × 256 pixel patches. Next, we extracted features from each patch using the transfer learning method in deep learning^34,35^. In this section, we used our fine-tuned and pre-trained DeepLab V3+ with ResNet-18 model^21^ to extract features for the clustering. Then, we used one of the most popular unsupervised machine learning algorithms called K-means clustering. All patches were clustered through K-means clustering to cluster similar image sub-regions together. Finally, a histogram representation for each biopsy sample was created to describe the distribution of each type of cluster at the patient level. We used a random forest model as a classifier to calculate AUC and predict association of clusters with clinical patient outcomes such as eGFR.

### Stain normalization

**Figure 2:**
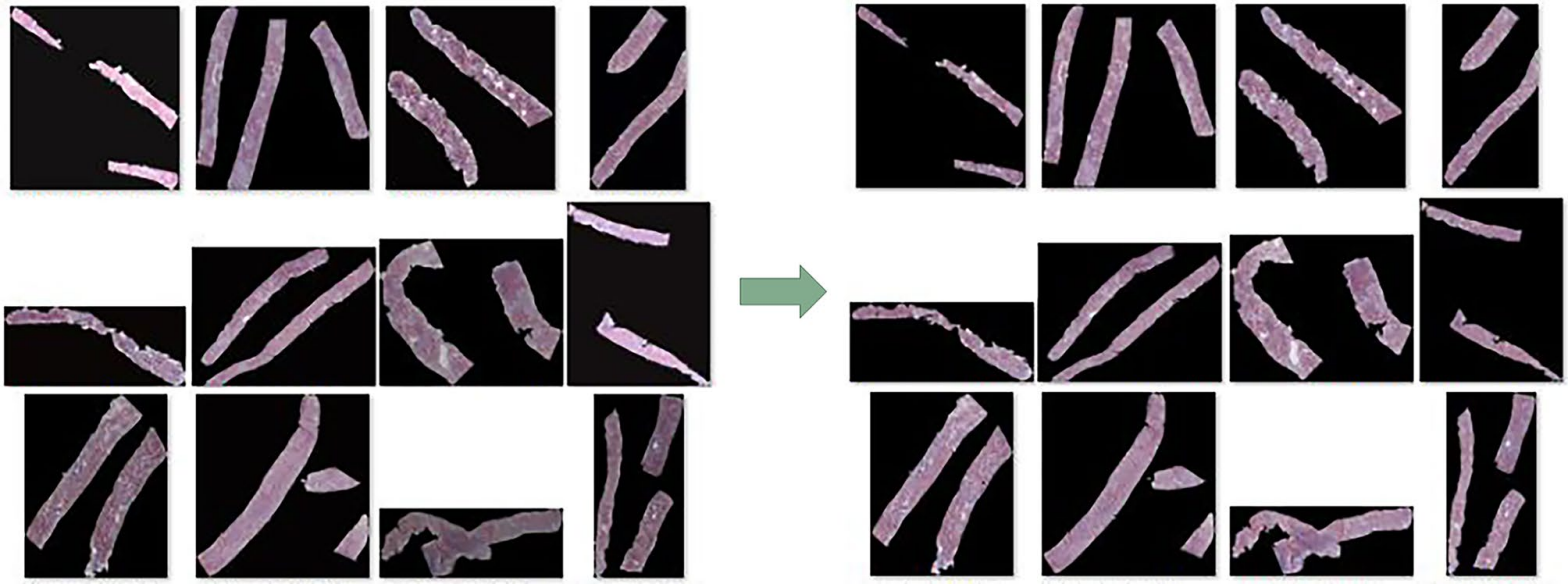
Example images of biopsy samples. Multiple cortexes are combined in each case. To reduce the color and intensity variations present in the stained images, we computed the global mean and standard deviation of each channel in the Lab color space for the Reinhard color normalization for all data and used them as reference values to normalize our data. The figure shows Reinhard color normalization before (left) and after (right).

Computer-aided techniques are affected by the variations in color and intensity of the images. In this study, we performed Reinhard color normalization on all whole-slide-imaging data as a preprocessing step to increase the computational efficiency and performance^33^. We computed the global mean and standard deviation of each channel in the Lab color space for the Reinhard color normalization for all data and used them as reference values to normalize our data. There are multiple biopsy samples for each case. Figure 2 shows an example of data with color stain normalized images.

### Transfer learning

In this study, we used one of the most popular machine learning methods called transfer learning for feature extraction. Transfer learning is especially popular in medical image analysis for deep learning where the data are not sufficient for training^34–36^. Transfer learning uses a pre-trained deep learning model where a model was developed for a task and reused as the starting point for a model on another related task^37^.

**Figure 3:**
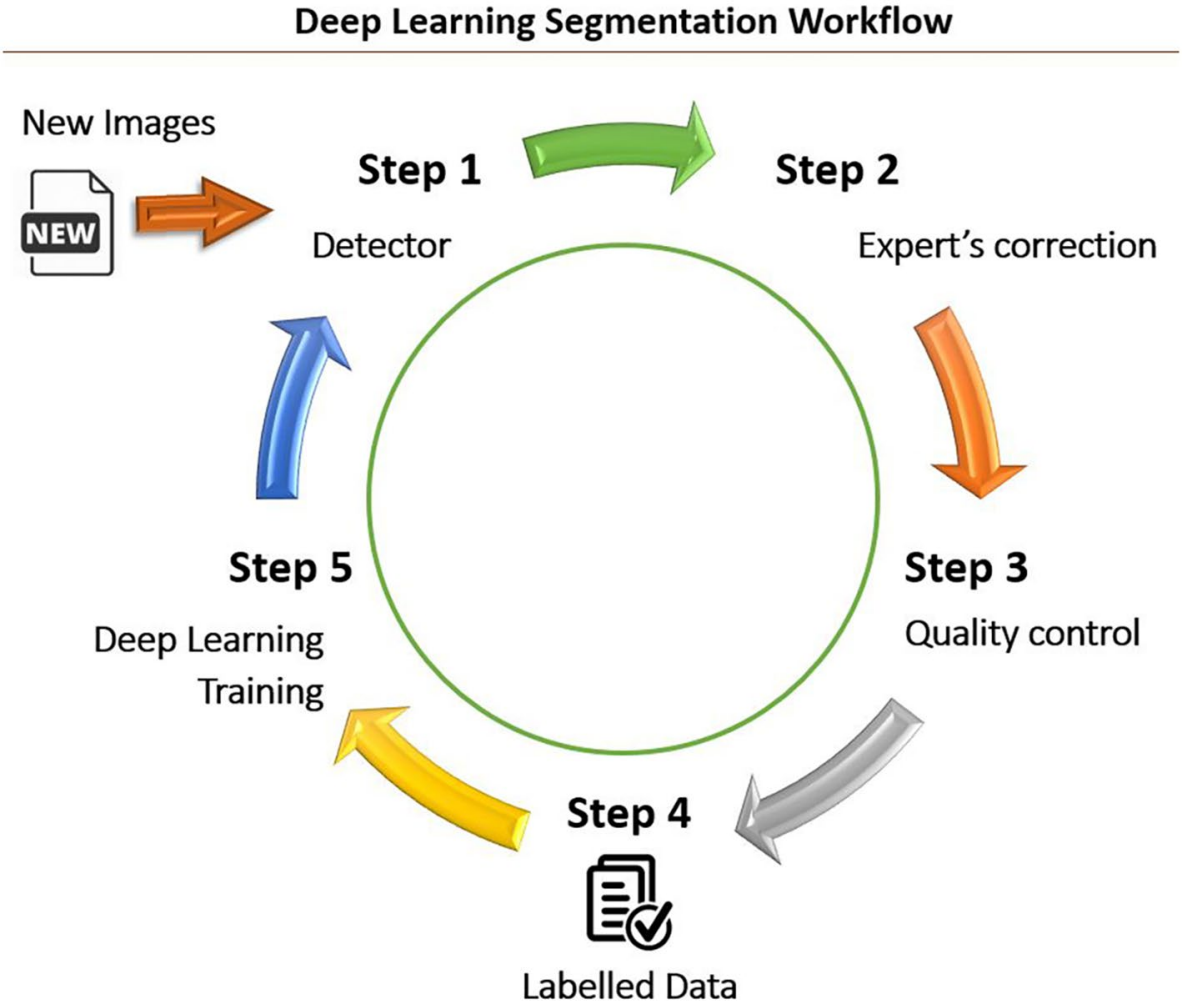
Workflow for the deep learning segmentation. Step 1: New images were fed into our model (detector) for automatic segmentation. Step 2: Our experts corrected errors manually. Step 3: These corrected segmented images were examined by a pathologist (JBH) for quality control. Step 4: Post-processing was performed to remove unwanted dots or pixels as errors. Step 5: The final gold standard labeled data was used to train our model to improve segmentation accuracy.

First, we used DeepLab V3+ with ResNet-18 architecture^21,38^ pre-trained on ImageNet^39^, a large image database that contains more than 14 million images with 20,000 categories. Then, we further trained this deep learning network for semantic segmentation on whole-slide images obtained from biopsy samples to automatically segment microscopic kidney structures. This additional training is called fine-tuning and it utilizes transfer learning to achieve better performance for our kidney structure segmentation. 136 images were selected randomly from whole-slide images with a size of approximately 3000 × 3000 pixels (720 µm × 720 µm). The data for this training was from the digital pathology image repository, C-PROBE cohort which is not used in the main analysis with 57 cases. We used predefined classes: open glomeruli, arterioles, globally sclerosed (GS) glomeruli, interstitium, and tubules. All remaining unannotated area, including artifact spots, were labeled as miscellaneous. The data was divided into the training set (60%), validation set (20%), and test set (20%). Each image (~ 3000 × 3000 with RGB channels) was tiled in non-overlapping 256 × 256 pixel patches. A total of 16,242 image patches were subsequently fed into the DeepLab V3+ to train the model. The workflow for segmenting kidney substructures is shown in Fig. 3. In the first step of the workflow, new images were fed into our model for automatic segmentation. In the second step, our experts identified errors and manually corrected them. In the third, a pathologist (JBH) examined the corrected images for quality control. Then, in the fourth step, post-processing was performed to remove unwanted dots or pixels as errors. In the fifth step, the final gold-standard labeled data was used to train our model to improve segmentation accuracy. The performance of our deep learning model for the multiclass segmentation was assessed on the test set.

### Patches and feature extraction

For our main analysis with 161 biopsy cores, each cortex was extracted and the background was removed manually from the WSI by a pathologist using QuPath^40^ and ImageJ^41^. Multiple cortexes were combined into one image for each case. After stain color normalization, each image (a size of about 20,000 × 20,000) was partitioned into smaller image patches with a size of 256 × 256 and a feature vector was extracted for each patch. A total of 107,471 feature vectors were extracted from all patches. We extracted features from a layer (res5b) of ResNet-18^42^, a decoder structure, which is a part of deep neural networks for semantic segmentation, DeepLab V3+.

### Unsupervised machine learning-clustering

In this study, we used K-means clustering which is one of the simplest and popular unsupervised machine learning algorithms. Generally, unsupervised learning algorithms make inferences from datasets using only input data without knowing labels or outcomes. Clustering algorithms form groupings using similarity or distance measure. Before performing the clustering algorithm, we used the algorithm Silhouette in MATLAB to find the optimal number of data clusters (K) in K-means clustering to group patches with similar visual features. Then, we performed K-means clustering and obtained cluster indices, centroid locations, and distances from each point to every centroid for the analysis. K-means clustering is one of the most popular unsupervised machine learning algorithms that aims to partition *n* observations into *k* clusters in which each observation belongs to the cluster with the nearest mean (cluster center or cluster centroid), serving as a prototype of the cluster.

### Bag-of-words and visual dictionary

In this study, we developed a methodology for image feature extraction based on a bag-of-words approach^19,31^ to find previously unknown features that predict patient outcomes. We built the visual dictionary that consists of representative visual words from each cluster (Fig. 6A) using the K-means clustering performed on the feature vectors for each image tile, obtained from all the images across the patients.

### Data analysis with histograms

The histogram representation for each tissue was then constructed in terms of the obtained clusters, visual words from K-means clustering (Fig. S1 in the Supplementary Information). This frequency (or occurrence) on the histogram representation will represent how often each unsupervised machine learned phenotype is encountered for each tissue. This frequency for each visual word was used as a feature for predicting patients’ estimated glomerular filtration rate (eGFR). In addition to the individual frequency or occurrence of visual words, we also used polynomial coefficient features that combine all frequencies for each case. This was done by using the multidimensional scaling (MDS) and polynomial fitting function. MDS allows us to calculate the dissimilarity between groups (visual words or phenotypes) with the Euclidean distances and visualize how near groups are to each other in histogram plots. Once the distance between groups was obtained and arranged in order of distance, we then applied the 4th polynomial fitting on the frequency histogram and obtained five coefficients for each case.

1 $$ f\left( x \right) = c_{1} x^{4} + c_{2} x^{3} + c_{3} x^{2} + c_{4} x^{1} + c_{5} $$

where *c*_*1*_*, c*_*2*_*, …, c*_*5*_ are the coefficients of the 4th polynomial function *f(x)*. In addition to the individual frequency or occurrence of visual words on the histogram, this polynomial fitting on the histogram (Fig. S1 in the Supplementary Information) provided overall information about all histogram frequency features. In this study, we used histogram frequency features, polynomial coefficient features, and clinical features such as age, race, gender, and diagnosis. The detailed clinical features are shown in Table S4 and Fig. S2 in the Supplementary Information. Patient diagnosis and demographics demonstrate a diverse cohort (Table 1). The patients were divided into two groups based on eGFR ≥ 60 (n = 36) and eGFR < 60 (n = 21) and we used a random forest algorithm as a classifier to predict dichotomized eGFR groups. We also identified the important visual words or morphologic features that associate with the level of kidney function at the biopsy in CKD patients. Also, we selected the top features for the analysis based on their rank of feature importance. We established this by using the Gini index, also known as Gini impurity, which calculates the amount of probability of a specific feature that is classified incorrectly when selected randomly. Gini index was computed by Eq. (2)

2 $$ G = 1 - \mathop \sum \limits_{i = 1}^{m} p_{i}^{2} $$

where *m* is number of classes and $p_{i}^{2}$ is the probability of picking a data point with class *i*.

**Table 1:** Baseline characteristics of the participants.

Diagnosis		Race		Gender	
0	Lupus (n = 22)	0	White/Caucasian (n = 38)	0	Male (n = 16)
1	Minimal change/FSGS (n = 8)	1	Black/African American (n = 11)	1	Female (n = 42)
2	Membranous nephropathy (n = 3)	2	Asian/Asian American (n = 4)		
3	IgA/HSP (n = 9)	3	Multiracial (n = 1)		
4	Other GN (n = 3)	4	American Indian/Alaskan Native (n = 1)		
5	Diabetic nephropathy /Hypertensive nephropathy (n = 12)	5	Others (n = 2)		

In addition, we applied our proposed methodology to predict whether eGFR is decreased or increased in one year. In order to do that, the patients were divided into two groups based on eGFR slope ≥ 0 (n = 30) and eGFR slope < 0 (n = 27) and we used a random forest algorithm as a classifier to predict dichotomized eGFR slope. The eGFR slope is defined as Eq. (3).

3 $$ eGFR\,slope = \frac{eGFR\,in\,year\,1 - eGFR\,at\, the\, biopsy}{{age\,at\,year\,1 - age\,at\,the \, biopsy}} $$

where “age at year 1” is the age in days approximately 1 year after the biopsy.

We performed a receiver operating characteristic (ROC) curve analysis, which is a graphical plot that illustrates the diagnostic ability of a binary classifier system. To evaluate the performance of our model, we estimated the area under the ROC curve (AUC) and its 95% confidence interval^43–45^. All data processing and analyses were done with MATLAB (R2020a, The MathWorks, Inc.) and R (R Foundation for Statistical Computing, Vienna, Austria).

## Results

**Figure 4:**
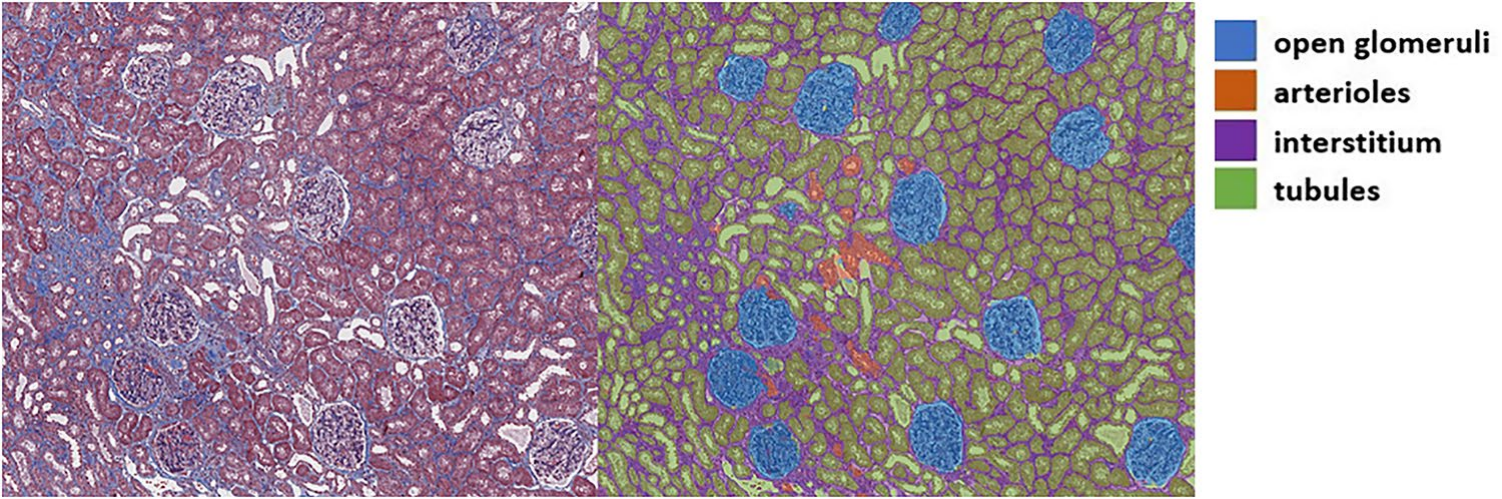
An example of a trichrome-stained image (left) and an automatically segmented image from our trained deep learning model (right).

First, we assessed the performance of our deep learning model for the multiclass segmentation on the test set (20% of 136 images). An example result of automatic segmentation by our deep learning model is shown in Fig. 4. The global accuracy was 0.95 and the highest accuracy for the individual class was 0.98 for tubules. The detailed results for 5 classes were summarized in Table 2.

**Table 2:** Deep learning segmentation results.

Structure	Glomeruli	Arterioles	GS Glomeruli	Interstitium	Tubules
Accuracy	0.95	0.87	0.88	0.91	0.98
IoU	0.92	0.78	0.75	0.84	0.77

GS, globally sclerosed; IoU, intersection over union.

**Figure 5:**
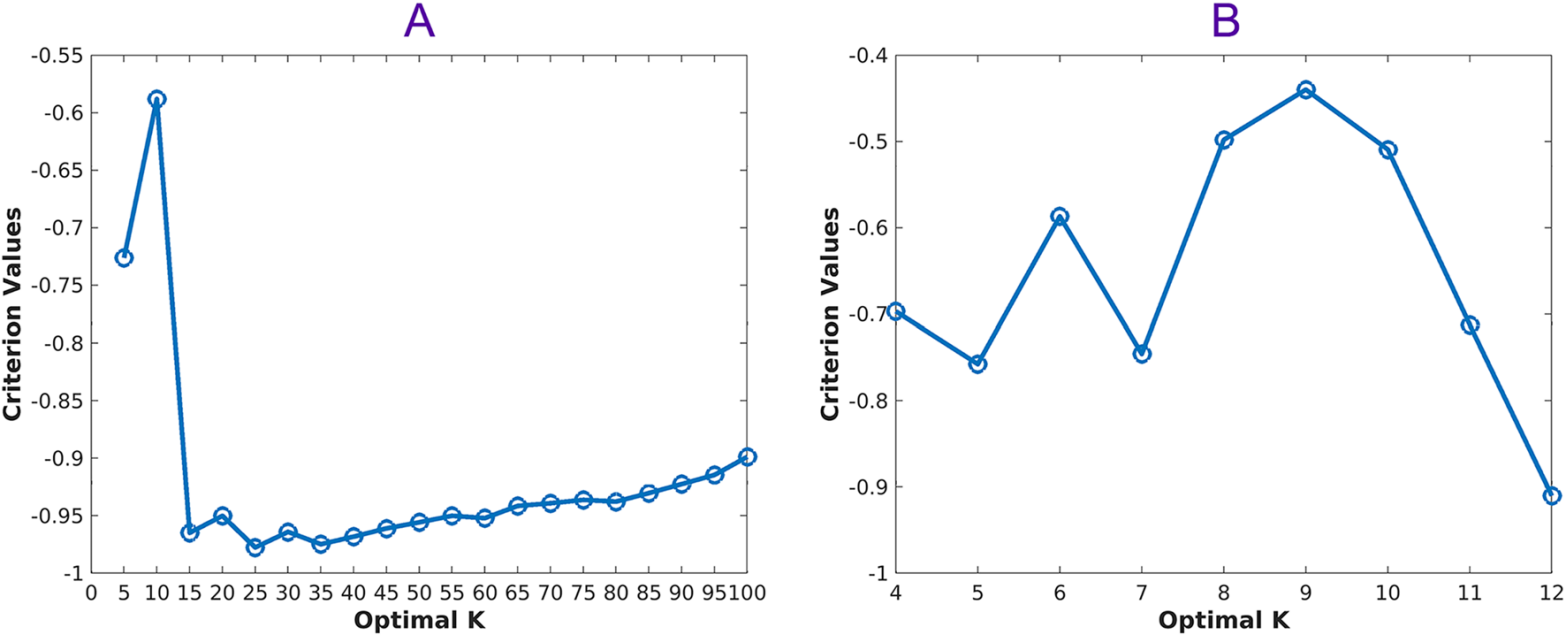
Optimal K using the Silhouette algorithm. (**A**) First, we run the algorithm every 5th point between 5 and 100 and then (**B**) run the algorithm between 4 and 12 to find the optimal K = 9 for the K means clustering.

**Figure 6:**
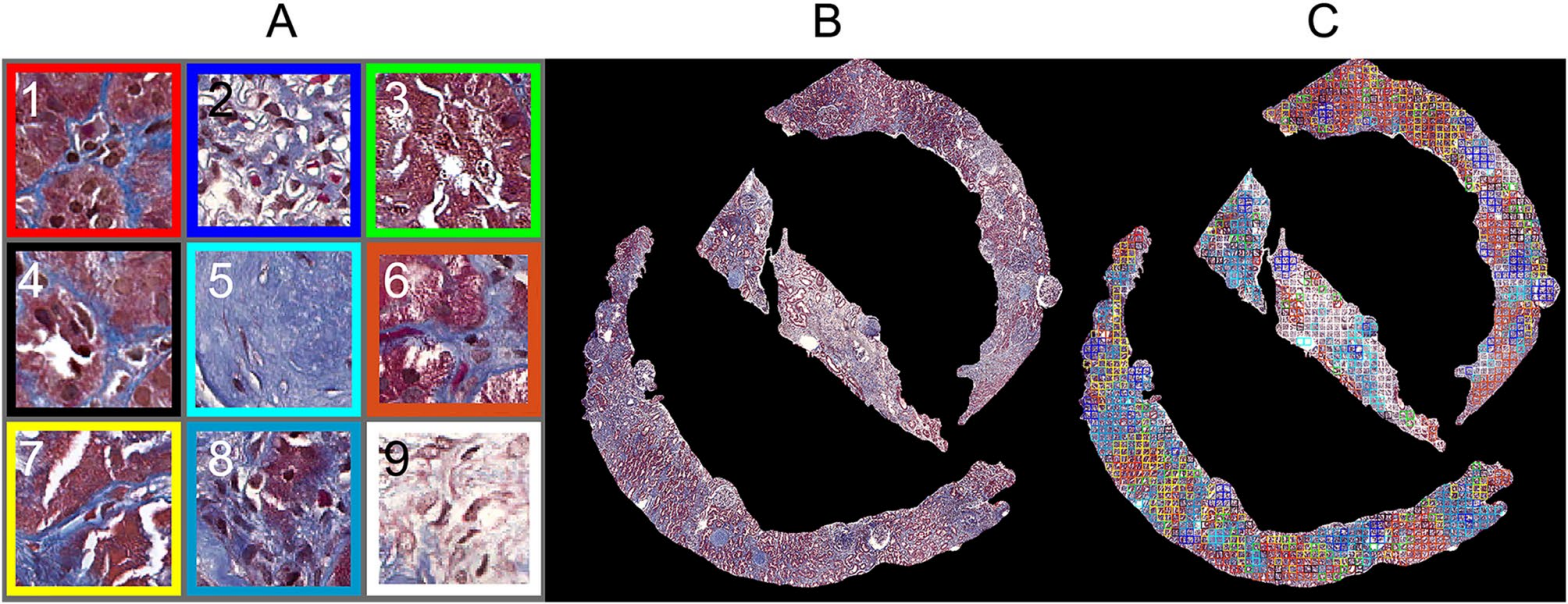
(**A**) A visual dictionary that consists of 9 representative visual words, (**B**) a representative cortex example, and (**C**) its cluster map with colored patches. Each colored patch corresponds to its assigned visual word.

**Figure 7:**
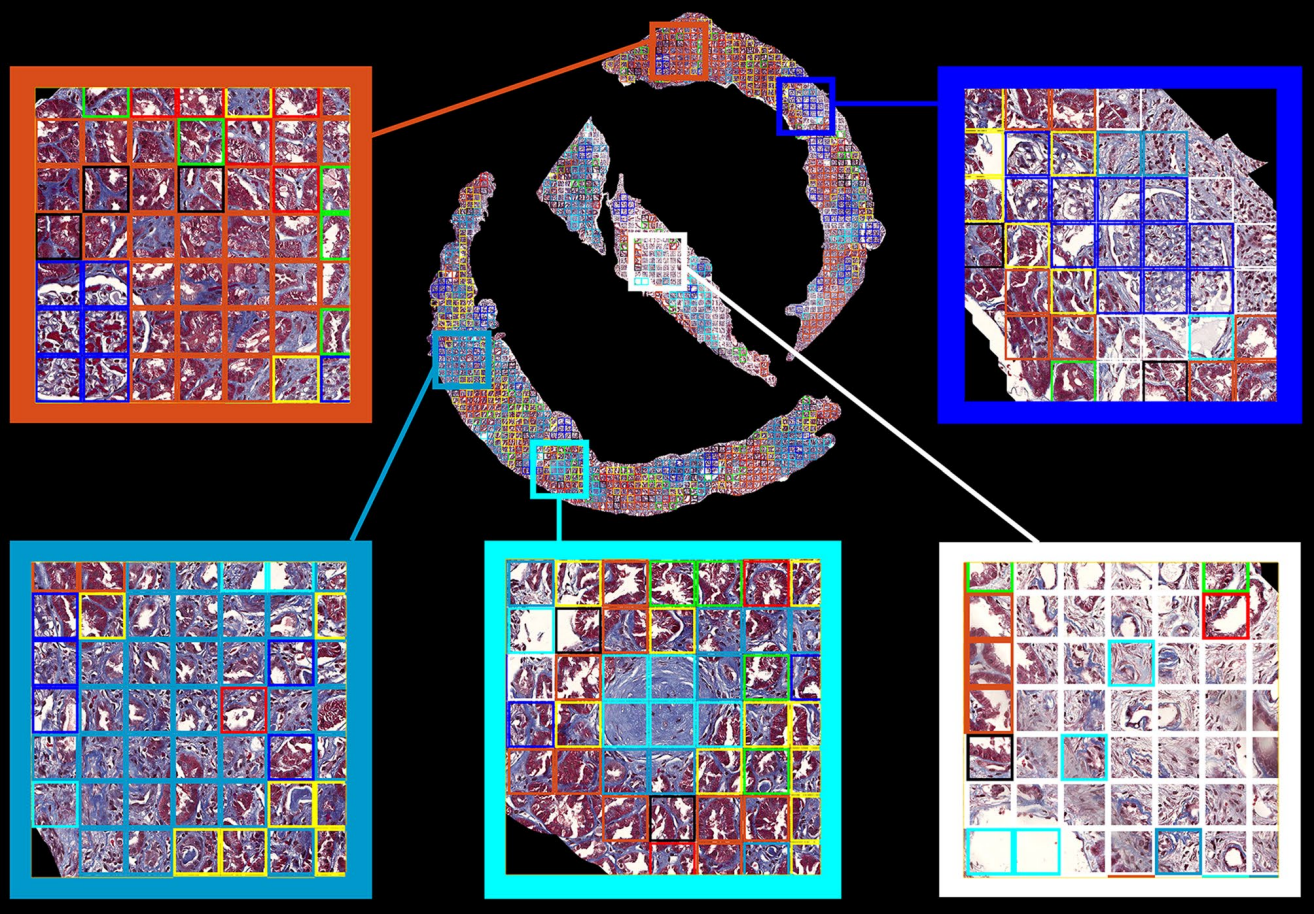
An example of cortex trichrome stained images with color-coded patches and zoomed images.

The optimal number of visual words (K = 9) using the algorithm Silhouette in K-means clustering is shown in Fig. 5. Next, we obtained cluster indices, centroid locations, and distances from each point to every centroid for the analysis with a K-means clustering method. Figure 6 shows (A) visual dictionary (9 representative visual words), (B) an example of cortexes, and (C) its cluster map and Fig. 7 shows zoomed images that contained visual words in each color boxes. The histograms with the 4th polynomial fitting plots for all 57 cases are shown in Fig. S1 in the Supplementary Information.

**Figure 8:**
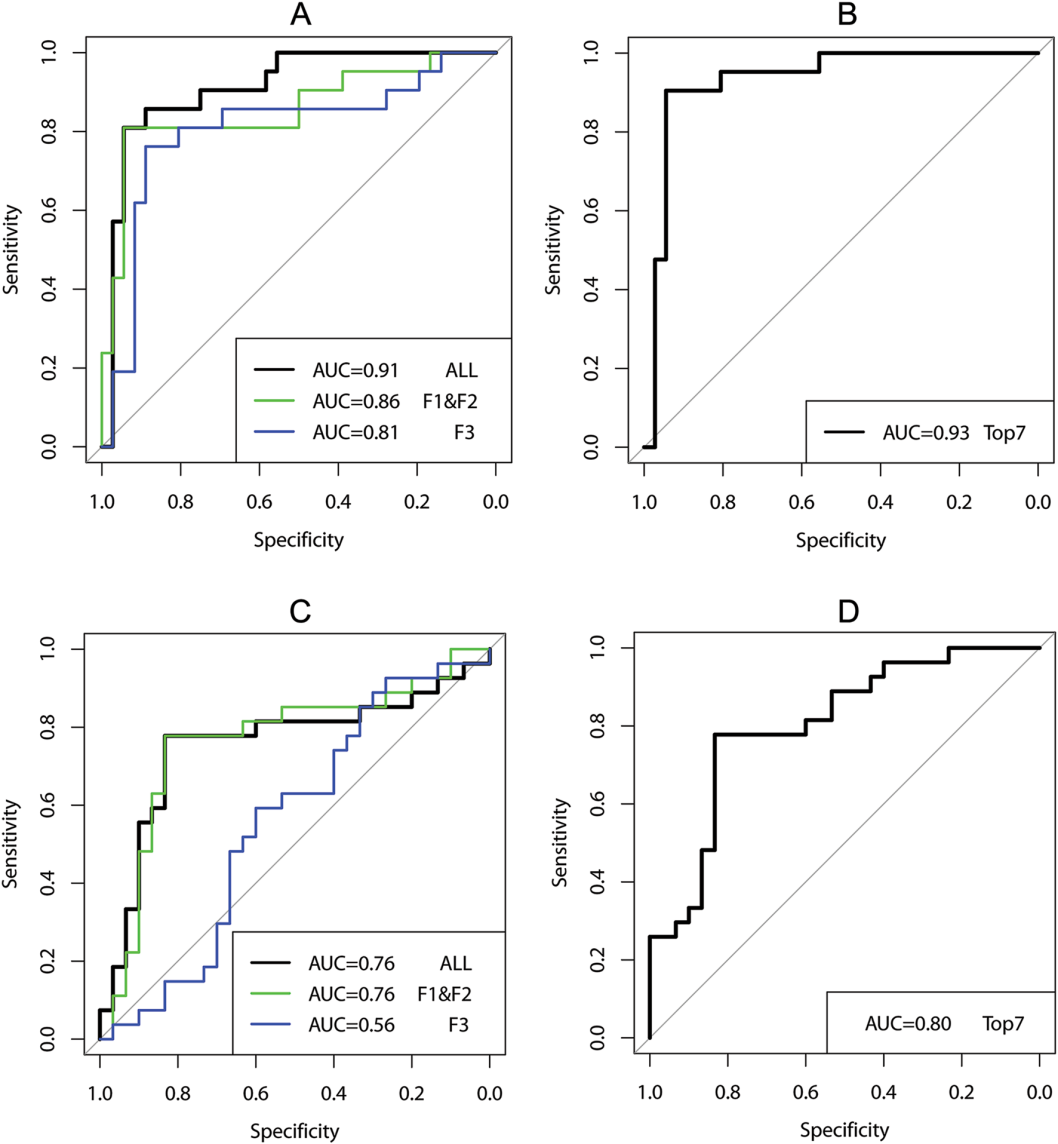
ROC curves for the prediction of the level of kidney function (**A**, **B**) at the biopsy and (**C**, **D**) in the future. F1, F2, and F3 represent frequency, polynomial fitting coefficients, and clinical features, respectively. Top7 represents the top 7 features selected based on the importance rank. The x-axis is the true negative rate (TNR) or specificity and the y-axis is the true positive rate (TPR) or sensitivity.

For predicting dichotomized eGFR at the biopsy, the error from the random forest was 0.11, the sensitivity was 0.94, and the specificity was 0.81. The ROCs are illustrated in Fig. 8. The x-axis represents the true negative rate (TNR) or specificity and the y-axis is the true positive rate (TPR) or sensitivity. The area under ROC curve (AUC) was 0.91 and the 95% confidence interval was 0.8322–0.9922. The accuracy was 0.89, calculated by using Eq. (4) for this model,

4 $$ ACC = \left( {TP + TN} \right)/\left( {TP + FN + TN + FP} \right) $$

where TP, FP, TN, and FN represent true-positive, false-positive, true-negative, and false-negative predictions, respectively. The detailed results are shown in Table S1 in the Supplementary Information.

In this study, with this unsupervised machine learning technique, we identified the important morphologic and clinical features associated with the level of kidney function in CKD patients both at the biopsy and in one year. The detailed rank for frequency features, polynomial coefficient features, and clinical features at the biopsy and in one year is shown in Tables 3 and 4, respectively. We established this by using the Gini index.

**Table 3:** Ranking of the important features for the dichotomized level of kidney function at the biopsy.

Features	Description	Gini index (importance)	Rank	Overall rank
Frequency (visual dictionary)	f1 (red)	0.64	9	16
	f2 (blue)	3.68	1	2
	f3 (green)	0.85	6	11
	f4 (black)	0.87	5	10
	f5 (cyan)	2.10	3	4
	f6 (orange)	0.69	8	15
	f7 (yellow)	1.26	4	7
	f8 (dark blue)	2.12	2	3
	f9 (white)	0.73	7	13
Polynomial coefficient	*c*_1_	1.69	1	6
	*c*_2_	1.17	2	8
	*c*_3_	1.09	3	9
	*c*_4_	0.70	5	14
	*c*_5_	0.85	4	12
Clinical	Age	4.68	1	1
	Gender	0.60	3	17
	Race	0.37	4	18
	Diagnosis	1.98	2	5

*c*_*n*_: polynomial coefficients in Eq. (1).

**Table 4:** Ranking of the important features for the prediction of eGFR slope.

Features	Description	Gini index (importance)	Rank	Overall rank
Frequency (visual dictionary)	f1 (red)	1.23	6	13
	f2 (blue)	1.21	7	14
	f3 (green)	1.55	5	11
	f4 (black)	1.96	4	4
	f5 (cyan)	0.72	9	17
	f6 (orange)	2.14	3	3
	f7 (yellow)	2.43	1	1
	f8 (dark blue)	2.27	2	2
	f9 (white)	0.75	8	16
Polynomial coefficient	*c*_1_	1.77	1	6
	*c*_2_	1.74	2	7
	*c*_3_	1.01	5	15
	*c*_4_	1.66	3	9
	*c*_5_	1.37	4	12
Clinical	Age	1.73	2	8
	Gender	0.13	6	20
	Race	0.24	5	19
	Diagnosis	0.67	4	18
	eGFR	1.82	1	5
	UPC	1.58	3	10

*c*_*n*_, polynomial coefficients in Eq. (1); UPC, urine protein creatinine ratio.

Visual words #2 and #8 were determined to be the most important visual words for determining the level of the kidney function at the biopsy stage (Table 3). While visual word #2 (blue) has morphological characteristics consistent with an open glomerulus, including both normal and inflamed regions, the morphological characteristics of visual word #8 (dark blue) are consistent with interstitial expansion, tubular atrophy, and some cellularity. The most important visual words for predicting the level of kidney function changes in one year are visual word #7 (yellow), with features consistent with normal and nearly-normal tubulointerstitial (TI) with more interstitial expansion, and visual word #8 (dark blue). Table 5 presents a detailed description for each visual word. We selected the top 7 features based on the important feature rank (Table 1); 4 frequency features (f5, f6, f8, and f9), 1 polynomial feature (c1), and 2 clinical features (age and diagnosis). Selecting the top 7 features ensured that all three categories of feature types were included in our analysis. The error from the random forest was 0.07, the sensitivity was 0.94, and specificity was 0.89. The ROC for the top 7 features is illustrated in Fig. 8 (A, right). The AUC was 0.93 and the 95% confidence interval was 0.8605–1.0. The accuracy was 0.93 (Table S2 in the Supplementary Information). For predicting whether eGFR is increased or decreased in one year (eGFR slope), the error from the random forest was 0.19, the sensitivity was 0.83, the specificity was 0.78, and the accuracy was 0.81. The ROC for the top 7 features is illustrated in Fig. 8 (B, right). The area under ROC curve (AUC) was 0.80 and the 95% confidence interval was 0.62–0.89 (Table S3 in the Supplementary Information). We also identified the important morphologic and clinical features associated with the prediction whether eGFR is increased or decreased in one year for CKD patients (Table 4). We also performed the classification with a random forest classifier 10 times to compute the mean and standard deviation of the OOB error for the top 7 features. For the prediction of eGFR at the biopsy, the average accuracy and standard deviation were 90.17 and 2.22, respectively. For the prediction of eGFR in one year, the average accuracy and standard deviation were 78.27 and 1.74, respectively (Figure S3).

**Table 5:** Description of 9 representative visual words.

Visual words	Corresponding kidney structures	Rank (at the biopsy)	Rank (slope)
#1 (red)	Normal TI	9	6
#2 (blue)	Open glomerulus including normal and inflamed but not GS	1	7
#3 (green)	Normal TI-more white space or cells	6	5
#4 (black)	Normal TI, some interstitial expansion	5	4
#5 (cyan)	GS, IF, Arterioles including white space	3	9
#6 (orange)	Normal TI	8	3
#7 (yellow)	Normal and nearly normal TI with more interstitial area	4	1
#8 (dark blue)	Mostly interstitial expansion and tubular atrophy and some cellularity	2	2
#9 (white)	Interstitial expansion	7	8

TI, tubulointerstitial; GS, glomerulosclerosis; IF, interstitial fibrosis.

## Discussion

We proposed an unsupervised machine learning method to cluster the image patterns or features of microscopic kidney structures in CKD and spatially encoded the original histopathology images using these words. Supervised learning uses machine learning algorithm that is defined by its use of labeled datasets. These datasets with corresponding labels or outcomes are designed to train (or supervise) algorithms into predicting their labels or outcomes. Unlike supervised learning, unsupervised learning uses machine learning algorithm to cluster datasets without using labels. Unsupervised machine learning methods can help us to discover previously unknown features that are useful for categorizing and predicting patient outcomes without human input. In this study, we constructed a predictive model to classify patients’ levels of kidney function with dichotomized eGFR at 60 as well as predicting whether eGFR is increased or decreased in one year.

Several studies have shown that artificial intelligence and machine learning methods are useful in solving diagnostic decision-making problems in CKD^23,27,46,47^. Xiao et al. investigated several statistical, machine learning, and neural network approaches for predicting the severity of CKD. These nine predictive models are logistic regression, Elastic Net, lasso regression, ridge regression, support vector machine, random forest, XGBoost, neural network, and k-nearest neighbor. They showed that the linear models including Elastic Net, lasso regression, ridge regression, and logistic regression have the highest overall predictive power with an average AUC and a precision above 0.87 and 0.80, respectively^46^.

To our knowledge, this is the first study in which unsupervised machine learning through visual bag-of-words has been used to cluster and identify important image patterns or morphologic features that are associated with the eGFR in CKD. In machine learning, there are two main types of learning methods, supervised and unsupervised learning. The main difference between the two methods is that supervised learning uses a ground truth or a prior knowledge of what the output should be. The goal of supervised learning is to approximate the mapping function so that the model can predict the correct label for new input data. Supervised learning problems can be further grouped as classification and regression problems. Most deep learning models today are supervised learning models which can be trained on a large supervised dataset, and each image has a corresponding label. On the other hand, unsupervised learning has no ground truth or correct answer and no labeled outputs. Unsupervised learning algorithms are left to their own devices to discover the patterns and structures in the data.

In this study, we have shown that unsupervised machine-learned features are potential surrogates of predicting eGFR and can be used as prognostic tools as well as for objective assessment of the level of kidney function in CKD. Our results demonstrate that the addition of visual words into a predictive model outperform clinical features alone. However, there are some limitations in our retrospective study. First, determining an optimal number of clusters in a dataset is a fundamental issue in k-means clustering. Partitioning clusters (e.g., k-means clustering) requires the user to specify the number of clusters before performing clustering algorithms, but there is no definitive answer as to the true number of clusters. We used one of the most popular algorithms, silhouette, to find an optimal number of clusters in k-means clustering. The silhouette method measures the quality of a clustering and its value indicates a measure of how similar an object is to its own cluster compared to other clusters^48^. However, the effects of the number of clusters on the clustering performance will be a subject of future study. Similarly, the effects of stain color normalization, the size of the tile, and various staining methods need to be examined more systematically in future studies. One issue regarding stain color normalization is that researchers commonly select a target or reference image randomly for the color normalization but this could lead to a significant bias in the results of the feature extraction and clustering. To avoid this issue in this study, we computed the global mean and standard deviation of all the WSI data as reference values to normalize our data. Our study included diverse etiologies of kidney disease. However, our study was interested in identifying structural features associated with CKD progression which were shared across disease etiologies and future work can be done within diagnostic categories. In addition, our current study mainly focused on the number of visual words or patterns presented in a case. The spatial pattern of fibrosis could be an important factor to be considered for the level of kidney function; however, because our method is not able to capture spatial information of visual words, this could be the reason for misclassified cases (Figure S4). The future study will add this spatial information or patterns to the analysis to improve the model performance.

We identified important visual dictionary or morphologic features with respect to the level of kidney disease both at the biopsy (Table 3) and in one year (Table 4) through this unsupervised machine learning technique. Morphological characteristics from the algorithm-derived visual words were validated as important clinical features in pathological analysis of biopsy tissue. For example, visual word #8 (dark blue) contains morphological characteristics consistent with interstitial expansion and tubular atrophy as well as some cellularity. For prediction of the level of kidney function changes in one year, the most important visual words are visual word #7 (yellow), which carries features consistent with normal and nearly normal tubulointerstitial (TI) with more interstitial expansion, and visual word #8 (dark blue). Notably, while visual word #2 (open glomerulus) is more important for classifying the level of kidney function at the biopsy, visual word #7 (normal and nearly normal TI) is more important for classifying the level of kidney function changes in one year.

For the polynomial coefficient features, based on our results (Table 1), the most important polynomial coefficient was *c*_*1*_, a leading coefficient, which is the coefficient of the highest-degree term of the polynomial function for both eGFR at the biopsy and eGFR slope. This leading coefficient tells us what direction of the fitting curve is facing and the ends of the curve line behavior. The coefficient features can give us overall information of the histogram frequency features and this leading coefficient contributed the most for this. Among clinical features, age is the most important feature for prediction at the biopsy stage, while eGFR and age are the most important features for prediction of kidney function changes in one year. In this study, we showed that unsupervised machine learning methods could help elicit previously unrecognized diagnostic, morphological features that are predictive of kidney function at the biopsy stage and in predicting future kidney function.

In digital pathology image analysis, obtaining gold standard labels of micro kidney structures is a very time-consuming task requiring experts’ efforts due to the large image size and high resolution. In fact, manual segmentation on WSIs is almost impossible. The major advantage of our study is that we developed a methodology that does not require labels to find previously unknown features that predict patient outcomes.

In this study, we have demonstrated the feasibility of using an unsupervised machine learning method without human input in order to predict the level of kidney function, or eGFR, in CKD. The results from our study indicate that the visual dictionary, or visual image pattern, obtained from unsupervised machine learning can predict outcomes using machine-derived values that correspond to both known and unknown clinically-relevant features. These morphological characteristics can not only predict current and future CKD status, but can also provide interpretability in the form of visualizations of predictive features. Our objective, data-driven approach way to identify such unknown features will be useful for discriminating levels of kidney function and could help in decision making during follow-up.

## Supplementary Information

Supplementary Information.

## Data Availability

The datasets generated during and/or analyzed during the current study are available from the corresponding author on reasonable request. Source codes and scripts are available at GitHub (https://github.com/aznetz/BoSVW).
