# Supplementary material for: Unsupervised machine learning for identifying important visual features through bag-of-words using histopathology data from chronic kidney disease
Source: Sci Rep. 2022 Mar 22;12:4832. doi: 10.1038/s41598-022-08974-8 (PMC8941143; doi:10.1038/s41598-022-08974-8)
Supplement: Supplementary file 1 — Supplementary Information. [file 41598_2022_8974_MOESM1_ESM.pdf]

Unsupervised machine learning for identifying important visual features through Bag-of-Words using histopathology data from Chronic Kidney Disease

Joonsang Lee[1], Elisa Warner[1], Salma Shaikhouni[3], Markus Bitzer[3], Matthias Kretzler[3], Debbie Gipson[4], Subramaniam Pennathur[3], Keith Bellovich[5], Zeenat Bhat[6], Crystal Gadegbeku[7], Susan Massengill[8], Kalyani Perumal[9], Jharna Saha[2], Yingbao Yang[2], Jinghui Luo,[2] Xin Zhang[1], Laura Mariani[3], Jeffrey B. Hodgin[2,*,†] & Arvind Rao[1,10,11,12,*,†], for the C-PROBE Study.

[1]Department of Computational Medicine and Bioinformatics, University of Michigan, Ann Arbor, MI, USA

[2]Department of Pathology, University of Michigan, Ann Arbor, MI, USA

[3]Department of Internal Medicine, Nephrology, University of Michigan, Ann Arbor, MI, USA

[4]Department of Pediatrics, Pediatric Nephrology, University of Michigan, Ann Arbor, MI, USA

[5]Department of Internal Medicine, Nephrology, St. Clair Nephrology Research, Detroit, MI, USA

[6]Department of Internal Medicine, Nephrology, Wayne State University, Detroit, MI, USA

[7]Department of Internal Medicine, Nephrology, Cleveland Clinic, Cleveland, OH, USA

[8]Department of Pediatrics, Pediatric Nephrology, Levine Children's Hospital, Charlotte, NC, USA

[9]Department of Internal Medicine, Nephrology, Department of JH Stroger Hospital, Chicago, IL, USA

[10]Department of Biostatistics, University of Michigan, Ann Arbor, MI, USA

[11]Department of Radiation Oncology, University of Michigan, Ann Arbor, MI, USA

[12]Department of Biomedical Engineering, University of Michigan, Ann Arbor, MI, USA

*****Correspondence and requests for materials should be addressed to J.B.H. (email: jhodgin@med.umich.edu) & A.R. (email: ukarvind@med.umich.edu)

†Both authors contributed equally to this work

Table S1. Confusion matrix and AUC for all features

		Prediction	
		0	1
Actual	0	34	2
	1	4	17
**Accuracy**		0.89	
**AUC**		0.91	
**95% CI**		0.83 – 0.99	

0: eGFR ≥ 60, **1:** eGFR < 60, **CI**: confidence interval

Table S2. Confusion matrix and AUC for the dichotomized eGFR for the top 7 features

		Prediction	
		0	1
Actual	0	34	2
	1	2	19
**Accuracy**		0.93	
**AUC**		0.93	
**95% CI**		0.86 – 1.0	

0: eGFR ≥ 60, **1:** eGFR < 60, **CI**: confidence interval

Table S3. Confusion matrix and AUC for the eGFR slope for the top 7 features

		Prediction	
		0	1
Actual	0	25	5
	1	6	21
**Accuracy**		0.81	
**AUC**		0.80	
**95% CI**		0.62 – 0.89	

0: eGFR slope < 0, **1:** eGFR slope ≥ 0, **CI**: confidence interval

Table S4. Clinical data for 57 CKD patients

Patients	Age	Gender	Race	Diagnosis	eGFR	UPC
1	33	Female	White/Caucasian	Lupus Class III	96.87	2.27
2	39	Female	White/Caucasian	Lupus Class V, MPGN	90.93	1.46
3	34	Female	White/Caucasian	Lupus Class V	118.92	1.9
4	32	Female	Black/African American	Lupus Class III, Lupus Class IV	113.06	0.43
5	24	Female	Asian/Asian American	Lupus Class III	121.27	1.87
6	49	Male	White/Caucasian	Minimal Change	70.58	7.71
7	23	Female	White/Caucasian	HSP	122.12	0.48
8	46	Male	White/Caucasian	FSGS, IgA Nephropathy	47.31	2.2
9	19	Female	American Indian/Alaskan Native	FSGS	119.68	0.9
10	66	Female	White/Caucasian	Diabetic nephropathy	26.62	4.6
11	68	Female	White/Caucasian	Minimal change, FSGS	27.63	3.36
12	20	Male	Black/African American	FSGS	70.82	1.27
13	5	Male	White/Caucasian	FSGS	118.9	1.86
14	41	Female	Black/African American	Lupus Class V, hypertensive nephropathy	43.59	0.15
15	51	Female	Black/African American	Lupus Class III, lupus Class V	98.93	8.25
16	40	Female	White/Caucasian	Lupus nephritis	79.98	5.37
17	11	Female	White/Caucasian	HSP	160.93	1.72
18	38	Female	White/Caucasian	IgA Nephropathy	93.52	5.97
19	22	Female	White/Caucasian	Lupus Class II, MPGN	104.65	2.69
20	37	Female	Black/African American	Lupus Class V	83.35	2.48
21	48	Female	White/Caucasian	Lupus Class III	107.78	2.9
22	58	Female	White/Caucasian	FSGS	32.67	0.21
23	55	Male	Asian/Asian American	IgA Nephropathy, Hypertensive nephropathy	34.4	2.95
24	91	Male	White/Caucasian	Glomerular disease	22.96	2.23
25	45	Female	White/Caucasian	Membranous Nephropathy	105.13	3.9
26	56	Male	White/Caucasian	Glomerular disease	96.94	0.08
27	19	Female	Black/African American	Lupus class IV	95.905	1.65
28	80	Male	White/Caucasian	FSGS, tubulointerstitial disease	30.8	8.13
29	26	Female	Multiracial	Lupus class III, Lupus class IV	142.74	0.73
30	6	Male	White/Caucasian	IgA Nephropathy	131.41	3.41
31	26	Female	Black/African American	Lupus Class V	117.93	0
32	20	Female	White/Caucasian	Lupus Class II	109.43	0.06
33	51	Female	White/Caucasian	Lupus Class III	85.36	2.69
34	29	Female	White/Caucasian	Lupus Class V	109.48	2.19
35	45	Female	#N/A	Lupus Class V	111.95	1.56
36	30	Female	Black/African American	Lupus Class V	73.94	4.51
37	73	Male	White/Caucasian	Diabetic nephropathy, hypertensive nephropathy	66.24	1.49
38	59	Male				
39	37	Female	White/Caucasian	Diabetic nephropathy, hypertensive nephropathy, Tubulointerstitial disease	19.52	9.11
40	53	Female	White/Caucasian	Diabetic nephropathy	15.75	1.39
41	58	Male	White/Caucasian	Diabetic nephropathy	56.05	0.91
42	42	Female	White/Caucasian	Diabetic nephropathy	44.68	6.74
43	69	Female	Black/African American	Diabetic nephropathy	64.23	1.01
44	62	Male	White/Caucasian	Diabetic nephropathy	18.29	6.49
45	21	Female	White/Caucasian	Lupus Class V	163.67	3.48
46	29	Female	White/Caucasian	Lupus Class IV	123.17	0.99
47	7	Female	Asian/Asian American	Membranous Nephropathy	180.4	3.19
48	62	Male	White/Caucasian	IgA Nephropathy	53.47	0.31
49	71	Male	White/Caucasian	FSGS	46.17	2.74
50	36	Female	#N/A	IgA Nephropathy	114.22	0.43
51	37	Female	Asian/Asian American	Glomerular disease	114	0.42
52	31	Female	White/Caucasian	FSGS, IgA Nephropathy	51.27	1.72
53	51	Female	Black/African American	Membranous Nephropathy	91.94	6.31
54	67	Male	Black/African American	Diabetic nephropathy, Tubulointerstitial disease	25.88	5.54
55	48	Female	White/Caucasian	Diabetic nephropathy, Lupus Class II	44.32	8.42
56	59	Female	White/Caucasian	Diabetic nephropathy	25.87	8.53
57	48	Male	White/Caucasian	Diabetic nephropathy, FSGS	48.72	0.53

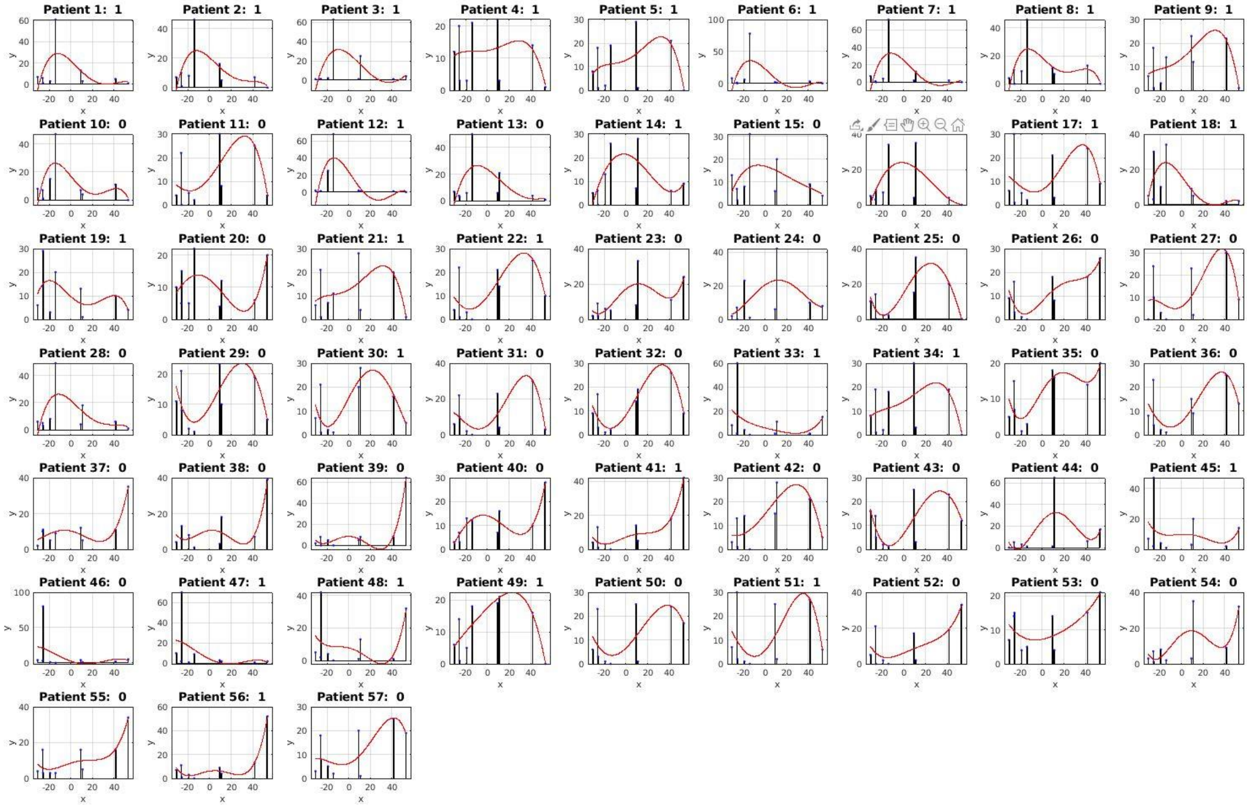

Figure S1. Frequency Histograms with the 4th polynomial fittings for all 57 cases with labels (0 for eGFR ≥ 60 and 1 for eGFR < 60). The x-axis represents the distance between cluster groups obtained from MDS and y-axis represents the normalized frequencies of the clusters.

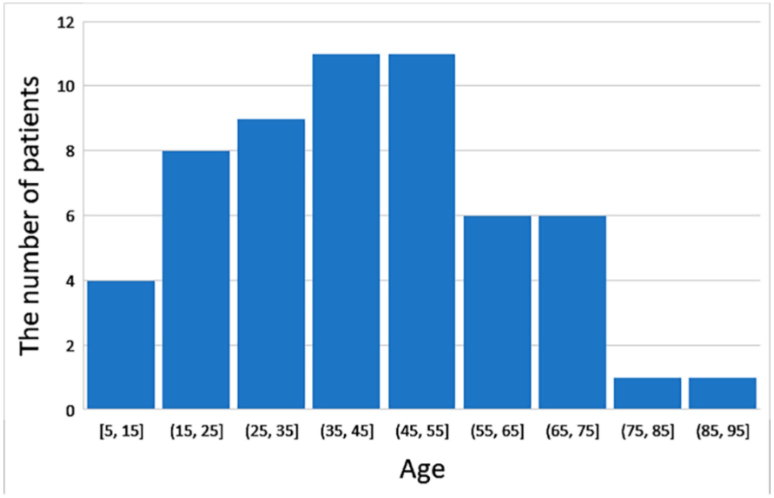

Figure S2. Histogram for the patients' age (ranged from 5 to 91)

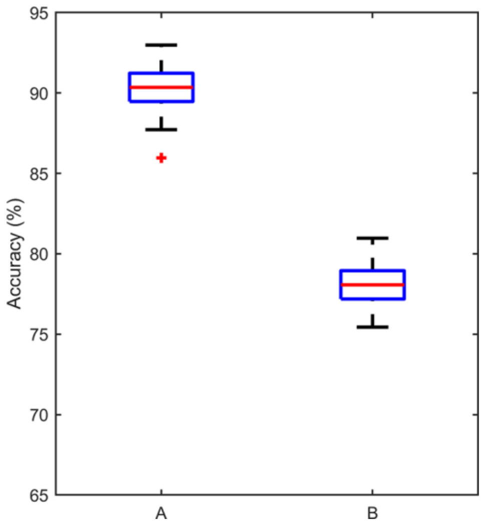

Figure S3. Boxplot for the mean accuracy and standard deviation. we performed the classification with a random forest classifier 10 times to compute the mean and standard deviation of the OOB error for the top 7 features. (A) For the prediction of eGFR at the biopsy, the average and standard deviation were 90.17 and 2.22, respectively. (B) For the prediction of eGFR in one year, the average accuracy and standard deviation were 78.27 and 1.74, respectively.

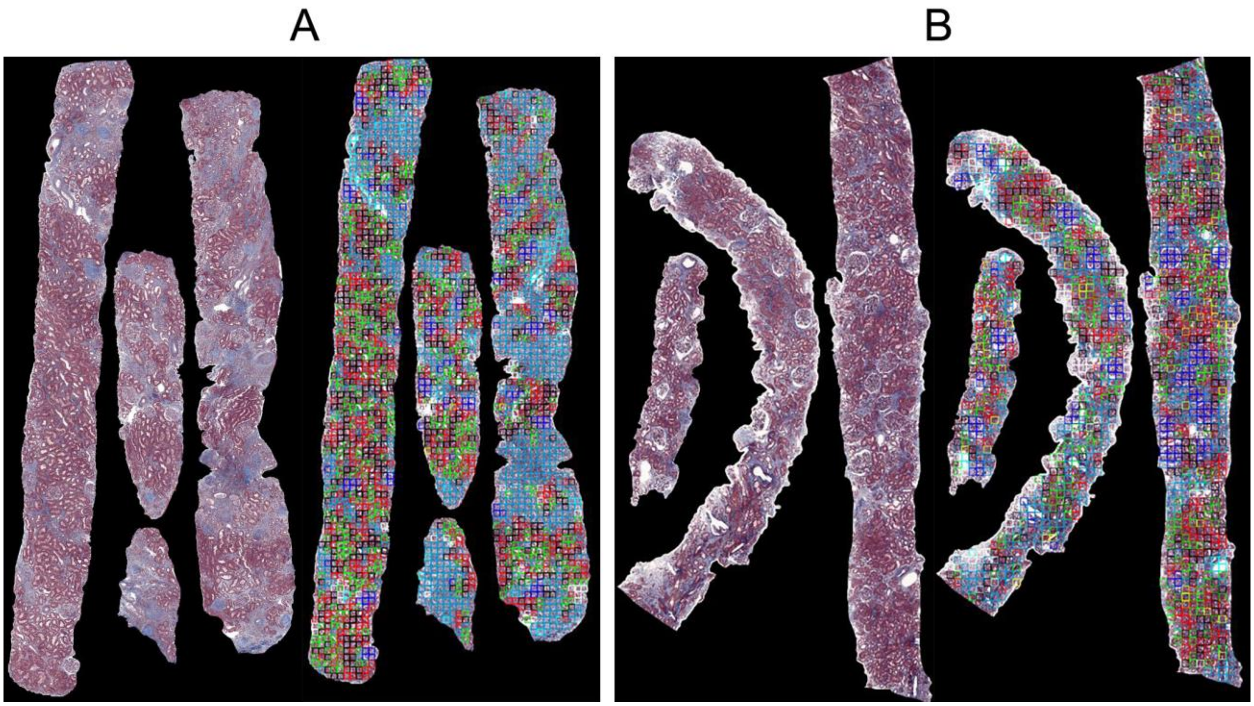

Figure S4. Examples of misclassified cases. (A) True eGFR = 49, predicted eGFR ≥ 60; (B) True eGFR = 97, predicted eGFR < 60
